# Correlation between expressions of Cyclin-D1, EGFR and p53 with chemoradiation response in patients of locally advanced oral squamous cell carcinoma

**DOI:** 10.1016/j.bbacli.2014.11.004

**Published:** 2014-11-21

**Authors:** Huma Khan, Seema Gupta, Nuzhat Husain, Sanjeev Misra, Negi MPS, Naseem Jamal, Ashim Ghatak

**Affiliations:** a Department of Radiotherapy, King George's Medical University, Lucknow, Uttar Pradesh, India; b Department of Pathology, RMLIMS, Lucknow, Uttar Pradesh, India; c Department of Surgical Oncology, King George's Medical University, Lucknow, Uttar Pradesh, India; d Clinical and Experimental Medicine Division, CSIR-Central Drug Research Institute, Lucknow, Uttar Pradesh, India

**Keywords:** Cyclin-D1, EGFR, p53 Immunohistochemistry, Squamous cell carcinoma, Chemoradiation

## Abstract

**Introduction:**

Cyclin-D1, p53 and EGFR are molecular markers that regulate the cell cycle and play an important role in tumor progression and development. The present study evaluates the prognostic significance of these markers with chemoradiation response in patients of locally advanced oral squamous cell carcinoma (OSCC).

**Material and method:**

A total of 97 OSCC patients (females = 19 and males = 78), aged 20–67 years and stage III/IV were recruited. Treatment response was assessed according to WHO criteria. Cyclin-D1, p53 and EGFR expressions in tumor tissue was estimated by immunohistochemical (IHC) method and quantified as percentage positive nuclei.

**Results:**

The positive expression rates of molecular markers were 86.6% for Cyclin-D1, 92.8% for EGFR and 85.6% for p53. The strong positive expressions of both Cyclin-D1 and p53 showed significant association with poor response. The Cox multivariate regression analysis showed coexpressions of Cyclin-D1 and p53 a significant and independent predictor of overall survival (OR = 1.90, 95% CI = 1.45–4.82, p = 0.046) after adjusting the demographic, clinicopathological and radiological response. The strong positive expressions of Cyclin-D1 and p53 and coexpressions of Cyclin-D1, EGFR and p53 showed significant (p < 0.05 or p < 0.01 or p < 0.001) and lower survival as compared to negative or moderate positive expressions and coexpressions, respectively.

**Conclusion:**

Expressions and coexpressions of Cyclin-D1 and p53 may serve as a prognostic marker in OSCC patients.

## Highlights

• Cyclin-D1, p53 and EGFR are molecular markers that regulate the cell cycle.
• Coexpressions of Cyclin-D1, EGFR & p53 serve as prognostic marker in advanced OSCC.
• p53 alone may serve as prognostic marker in patients of locally advanced OSCC.

## 1 Introduction

Oral squamous cell carcinoma is the sixth most frequent cancer worldwide. It is a major cause of morbidity and mortality in developing nations, comprising up to 50% of all malignancies [1], [2]. In India a large fraction of cases occurs in males in their productive years of life. Majority of the cases present in advanced stages, likely related to the poorer treatment outcome [3].

It emanates from the fact that the clinical course of disease and treatment outcome can also vary in patients with primary tumor from same site, size and stage, which would be possibly due to poor monitoring of oral squamous cell carcinoma (OSCC) in the absence of reliable biomarkers [4], [5].

Hence a better understanding of the molecular mechanisms and identification of potential oncogenes in oral cancer may provide more accurate and useful prognostic markers and eventually help us in achieving the ultimate goal of delivering customized treatment to increase survival at the cost of minimal toxicity which enables the patient in leading a more productive disease free life [6], [7], [8].

Various tumor markers Cyclin-D1, p53, EGFR known to be inhibitors of apoptosis play crucial roles in the initiation of intracellular signaling pathways which regulate the activation of cell proliferation, invasion, angiogenesis, metastasis and thereby influence treatment outcome [9], [10], [11].

Expressions of these proteins have also been correlated with a more aggressive phenotype and worse prognosis; nevertheless its significance in terms of clinical response and survival has already been examined in few studies and needs to be further delineated for better treatment outcome [11], [12], [13].

Therefore, identification of suitable marker that could provide prognostic assessment of the disease and would help in designing more appropriate and effective treatment strategies for OSCC is warranted, so that limited resources available to patients can be conserved and undue treatment can be avoided.

The current study is hence proposed to assess the combined expressions of Cyclin-D1, EGFR and p53 and its prognostic significance with treatment response in oral cancer patients undergoing chemoradiation.

## 2 Material and method

A total of 97 histologically proven cases of locally advanced stages (III, IV) oral cancer with W.H.O. performance status of grade 0/1 attending radiotherapy O.P.D. at K.G. Medical University, Lucknow (UP), India, in the years 2009–2012 were enrolled in the study. These cases were assessed thoroughly (history, clinical examination and investigations). The study was approved by the ethics committee of the K.G. Medical University, and written informed consent was obtained from all patients before enrollment.

All the patients were given 2 cycles of induction taxol (175 mg/m^2^ day 1) and cisplatin (50 mg/m^2^ day 2) chemotherapy and were subjected for radiation along with concurrent cisplatin (35 mg/m^2^) 4-weeks from the completion of induction chemotherapy. Radiotherapy was given by External beam Conventional Method (200 CGy/fraction to a total dose of 70 Gy in 35 fractions in 7 weeks by cobalt^60^ to primary tumor site and neck.

The protocol plan was continued despite mucositis or dermatitis. However, the dose of cisplatin was reduced to 50% if the calculated creatinine clearance level was 30–50 ml/min. No cisplatin was given if the creatinine clearance level was less than 30 ml/min. In the presence of myelosuppression (WBC count < 4000/mm^3^ or platelets count less than 100,000/mm^3^), persistent fever that exceeded 38 °C or other clinically apparent infections, chemoradiation was postponed for 1 week or interrupted.

For histopathological and immunohistochemical studies, tumor samples from the lesion site were fixed in 10% buffered formalin and then embedded in paraffin. Paraffin embedded formalin fixed tissues were processed and routine H and E stained sections were evaluated to confirm the diagnosis of squamous cell carcinoma and to grade the lesion. Further sections were processed for Cyclin-D1, EGFR and p53 biomarkers by immunohistochemistry (IHC) using primary monoclonal antibodies and a polymer based secondary antibody detection kit from Dakopatts, Denmark. Standard immunohistochemistry protocol was used. In short deparaffinized rehydrated sections were blocked for endogenous peroxidases in 0.3% hydrogen peroxide in methanol, followed by a rinse in distil water. Antigen retrieval was achieved at 121 °C in 10 mM citrate buffer (pH 6.0) for 10 min using Pascal retrieval system from Dakopatts, Denmark. Slides cooled to room temperature were washed thrice with TBS and thereafter incubated overnight at 4 °C with Primary Antibodies to Cyclin-D1 (Dakopatts Denmark), p53 (DO7, Leica Microsystems, Germany) and EGFR (BioGenex, USA). After washing with Tris-buffered saline, the sections were incubated for 30 min with secondary antibody. Cyclin-D1, EGFR and p53 were visualized with DAKO Liquid Diaminobenzidine substrate chromogen and counterstained with diluted Mayer's hematoxylin. Sections mounted with DPX were inspected under a Zeiss Z2 imager and photographed at 40× magnification.

The immunohistochemical evaluation was carried out in  tumor hotspots including the invasion front, which was regarded as most indicative of the biological activity of the tumor, in 10 high power fields. About 1500–2000 tumor cells were observed in all tumors at a magnification of 40× in 10 selected fields. For EGFR and Cyclin-D1 tumors were labeled as negative if < 10%, moderate positive between 10 and 50% and strongly positive if > 50%, tumor cells expressed the antigen [14], [15]. p53 expression was evaluated as negative if < 10%, moderate positive between 10 and 25% and strongly positive if > 25% [16].

Assessment of tumor response was done by clinical examination, radiological investigations (CT-scan) 4–6 weeks after completion of treatment. Biopsy or fine needle aspiration cytology to determine pathological response was not performed routinely; it was done only in the case of partial response/suspected lesion to confirm the presence of disease. After chemoradiation, patients were followed up to 2 years.

The definitions of treatment response viz. complete response (CR), partial response (PR) and no response (NR) [stable disease (SD) + progressive disease (PD)] were based on the standard definitions established by World Health Organization [33].

The end point was to evaluate clinical benefits of chemoradiation on response rate, 2 year overall survival (OS) and prognostic significance of Cyclin-D1, EGFR and p53 expressions with OS in locally advanced squamous cell carcinoma of oral cavity.

### 2.1 Statistical analysis

Continuous data were summarized as Mean ± SD while discrete (categorical) in no and %. Categorical groups were compared by chi-square (*χ*^2^) test. Groups were also compared by one way analysis of variance (ANOVA) and the significance of the mean difference between the groups was done by Tukey's post hoc test. Cox's univariate and multivariate hazard regression analyses were done to assess the predictors of overall survival. Survival between the two groups was compared by a Kaplan–Meier method using Log-rank test. A two-sided (*α* = 2) p < 0.05 was considered statistically significant.

## 3 Results

### 3.1 Basic characteristics of OSCC patients

The basic characteristics (demographic and clinico-pathological) of OSCC patients at presentation are summarized in Table 1. The age of patients ranged from 20–67 years with mean (± SD) 50.09 ± 12.15 years. Among patients, mostly 41–60 years aged (47.4%), mostly males (80.4%), mostly had lesion at buccal mucosa (24.7%) and mostly with poor performance status (56.7%). The histology of most of the patients was squamous cell carcinoma (64.9%), mostly with well differentiated grade (67.0%), tumor size T4 (75.3%), nodal status N2 (34.0%) and tumor stage IV (71.1%).

### 3.2 Molecular marker expressions and correlation

**Fig. 1:**
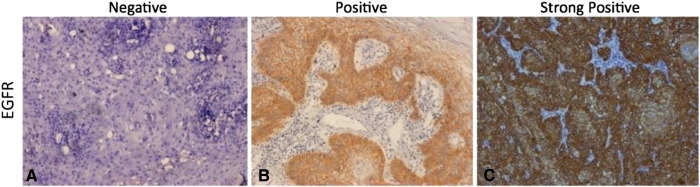
Microphotograph showing immunohistochemical expression of Cyclin-D1 in OSCC (A) showing negative nuclei (B) showing positive stained nuclei (C) showing strongly positive stained nuclei ( DAB × 125 × digital magnification).

**Fig. 2:**
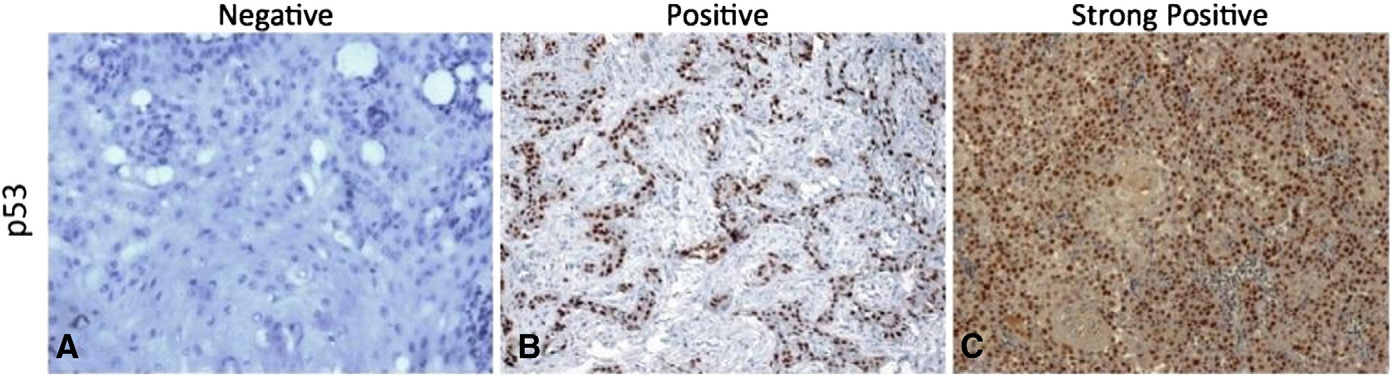
Microphotograph showing immunohistochemical expression of EGFR in OSCC (A) showing negative cytoplasmic and membranous staining (B) showing positive cytoplasmic and membranous staining (C) showing strongly positive cytoplasmic and membranous staining (DAB × 125 × digital magnification).

**Fig. 3:**
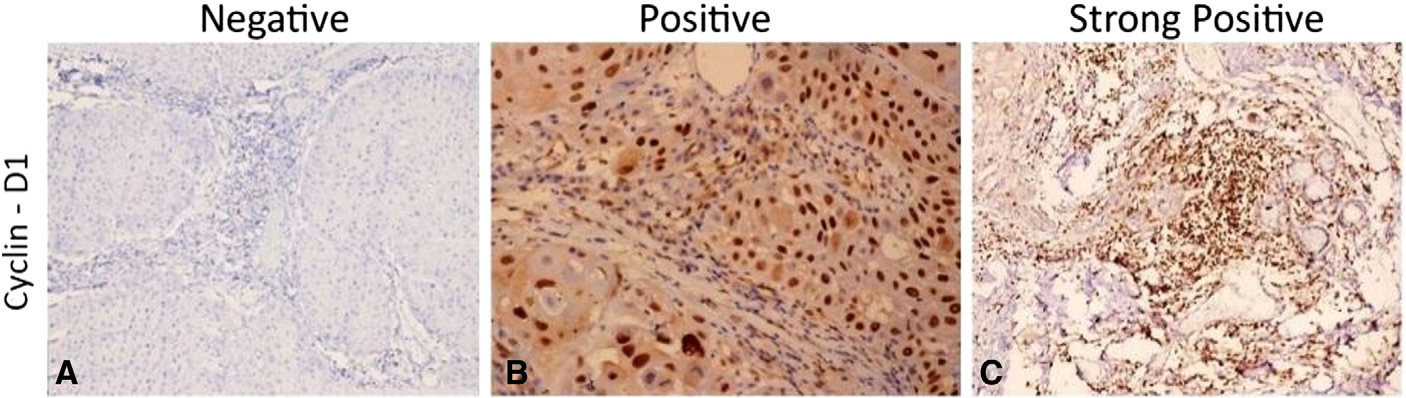
Microphotograph showing immunohistochemical expression of Cyclin-D1 in OSCC (A) showing negative nuclei (B) showing positive stained nuclei (C) showing strongly positive stained nuclei (DAB × 125 ×  digital magnification).

The immunohistochemical expressions of molecular markers EGFR, p53 and Cyclin-D1 were shown in Fig. 1, Fig. 2, Fig. 3 respectively. The expressions of molecular markers and their correlation in OSCC patients are summarized in Table 2, Table 3, respectively. The positive expression rates of molecular markers were 86.6% for Cyclin-D1, 92.8% for EGFR and 85.6% for p53 (Table 2). Further, molecular marker expressions of Cyclin-D1 and p53 showed a significant correlation with each other (*χ*^2^ = 29.27, p < 0.001); however, no statistically significant association was found between Cyclin-D1 and EGFR (*χ*^2^ = 2.56, p = 0.634) and EGFR and p53 (*χ*^2^ = 8.59, p = 0.072) (Table 3).

### 3.3 Association of molecular marker expressions with clinicopathological features

The associations between molecular marker expressions and clinicopathological features of OSCC patients are summarized in Table 4. The molecular marker expressions of both Cyclin-D1 (*χ*^2^ = 6.39, p = 0.041) and p53 (*χ*^2^ = 8.20, p = 0.017) showed significant association with nodal status. However, molecular marker expressions did not (p > 0.05) show significant associations with grade, tumor size and stage.

### 3.4 Association between molecular marker expression levels and radiological response

**Fig. 4:**
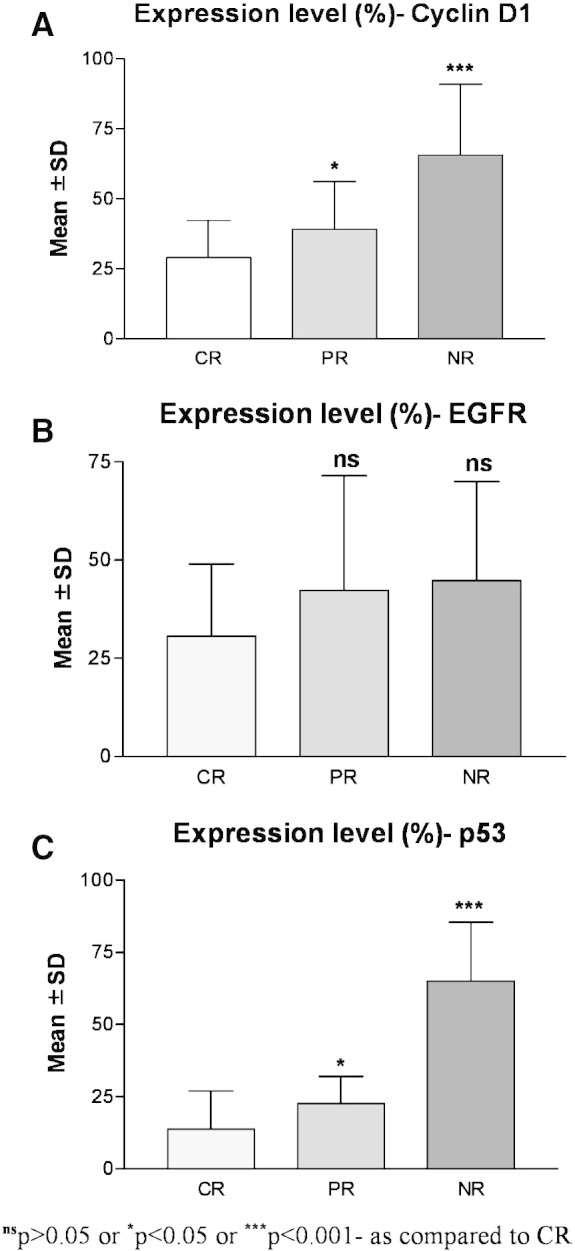
Association between molecular marker expression levels and radiological response in OSCC patients (A) showing association of NR with strong positive expressions of Cyclin-D1 along with higher mean expression levels of Cyclin-D1 in both PR and NR as compared with CR (B) showing association of PR and NR with an increase in levels of EGFR expression as compared with CR (C) showing a significant association of NR with strong positive expressions of p53 along with higher mean expression levels of p53 in both PR and NR as compared with CR.

The association between molecular marker expression levels (%) and radiological response is summarized graphically in Fig. 4. According to radiological response, 30 (30.9%) patients had complete response (CR), 51 (52.6%) had partial response (PR) and 16 (16.5%) had no response (NR).

Fig. 4 showed that as mean molecular marker expression levels increase, radiological response becomes poorer. Comparing the expression levels among the radiological response groups, ANOVA revealed significantly different expression levels of Cyclin-D1 (F = 22.85, p < 0.001) and p53 (F = 87.16, p < 0.001) among the groups. Further, Tukey's test also revealed significantly (p < 0.05 or p < 0.001) different and higher mean expression levels of both Cyclin-D1 and p53 in both PR and NR as compared with CR. Furthermore, the mean expression levels of both Cyclin-D1 and p53 were also significantly (p < 0.001) different and higher in NR as compared with PR. However, the mean expression level of EGFR did not show a significant association with radiological response.

Further, strong positive expressions of both Cyclin-D1 (*χ*^2^ = 27.92, p < 0.001) and p53 (*χ*^2^ = 69.40, p < 0.001) also showed a significant association with a poor response while strong positive expression of EGFR showed a significant association with a partial response (*χ*^2^ = 12.44, p = 0.014) (Table 5).

### 3.5 Association of molecular marker expressions and coexpressions with survivals

The OSCC patients were followed up for two years (24 months). During the period, 19 patients died due to disease (19.6%), 67 were live (69.1%) and 11 left the treatment (11.3%). The prevalence of live (live + LTF) patients was 80.4%.

The univariate (unadjusted) Cox regression analysis found molecular marker expressions of both Cyclin-D1 and p53 and coexpressions of Cyclin-D1 and p53, and EGFR and p53 the significant (p < 0.05 or p < 0.01) predictors of overall survival in OSCC patients (Table 6). The multivariate (adjusted) Cox regression analysis revealed the coexpressions of Cyclin-D1 and p53 a significant (p < 0.05) and independent predictor of overall survival in OSCC patients after adjusting the demographic (age, sex and performance status), clinicopathological features (site, histology, grade, size, nodal status and stage) and radiological response (Table 6).

**Fig. 5:**
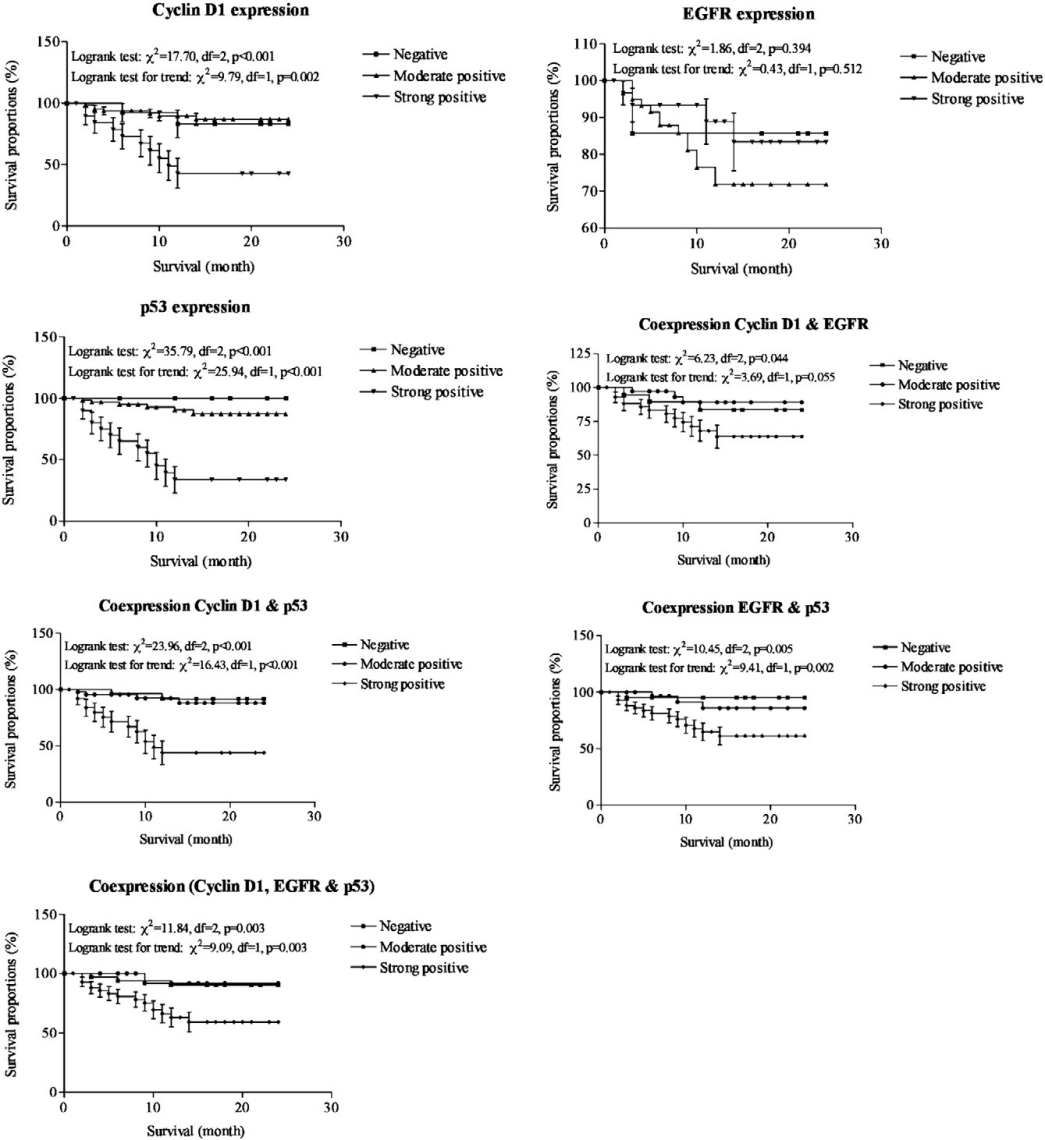
Overall survival proportions of OSCC patients according to marker expressions and coexpressions showing association of strong positive expressions of Cyclin-D1, EGFR, P53 and coexpressions of Cyclin-D1/EGFR, Cyclin-D1/p53, EGFR/ p53 and Cyclin-D1/EGFR/ p53 with lower survivals as compared with negative or moderate positive expressions and coexpressions.

The two year overall survival of OSCC patients was also done according to molecular marker expressions and coexpressions and summarized graphically in Fig. 5. The strong positive expressions of both Cyclin-D1 (*χ*^2^ = 17.70, p < 0.001) and p53 (*χ*^2^ = 35.79, p < 0.001) showed significant and lower survivals as compared with negative or moderate positive expressions. Further, the strong positive coexpressions of Cyclin-D1 and EGFR (*χ*^2^ = 6.23, p = 0.044), Cyclin-D1 and p53 (*χ*^2^ = 23.96, p < 0.001), and EGFR and p53 (*χ*^2^ = 10.45, p < 0.005) also showed significant and lower survivals as compared with negative or moderate positive coexpressions. Furthermore, the strong positive coexpressions of all three markers also showed a significant and lower survival as compared with negative or moderate positive coexpressions (*χ*^2^ = 11.84, p = 0.003).

## 4 Discussion

In this study, immunohistochemistry was used to evaluate Cyclin-D1, EGFR and p53 expressions in oral cancer. Chemoradiation is the basis of treatment in oral cancer worldwide, but the overall response rate is only about 30%, which may vary among individuals [17]. The study of cancer biology of OSCC can help in the molecular profiling of tumor markers that might predict the clinical behavior of the tumor, which is not strictly related to stage or histological grading as it is still not clear why some patients do better than others with the same stage and site of disease.

Cyclins, cyclin-dependent kinases, GSK3, cyclin-dependent kinase inhibitors and AKT1, AKT2, AKT3 kinases have emerged as critical mediators of signal transduction pathways downstream of activated tyrosine kinases and phosphatidylinositol 3-kinase are involved in the regulation of cell cycle progression and prevention of apoptosis. These are known to be associated with tumor genesis and resistance to apoptosis making the tumor refractory to treatment [18], [19], [20]. Various studies have reported a positive correlation between Cyclin-D1 protein expression and stage, lymph node involvement and reduced survival but did not reach statistical significance [21]. In this study, Cyclin-D1 expression in oral cancer was observed 92%, which is higher than the 19% reported for head and neck cancer which could be the potential targets for overcoming the treatment resistance.

Elevated EGFR expression in oral squamous cell carcinomas have also been associated with larger tumor and advanced stage and hence, a poor prognosis [22], [23]. EGFR activation by ligand binding leads to parallel signaling mainly through Ras, MAPK/MAPK-extra cellular signal regulated kinase, phosphatidylinositol-3 kinase/AKT phospholipase protein kinase α and signal transducer and activator 3 pathways [24], [25]. Activation of these pathways ultimately leads to transcription of other genes responsible for cell growth, differentiation and death. EGFR overexpression and aberrant EGFR gene copy number have been associated with poorer prognosis and disease-specific survival in SCCHN. Our study has also shown significance of EGFR as a high risk indicator for disease progression and propagation.

Among the genes related to oral cancer, p53 has been one of the most frequently studied. Abnormal p53 function has been detected in 33–100% of head and neck cancer specimens, depending on the sources of tissue and method of detection. p53 may participate in cellular pathways leading to apoptosis following treatment with DNA-damaging agents such as cisplatin [26]. Our data indicate that abnormal overexpression of p53 protein is a strong indicator of poor prognosis. Similar data have been demonstrated in bladder cancer where abnormal p53 overexpression has also been found to be associated with decreased survival [24].

Mutations of the tumor suppressor gene p53 are the most significant events in several human cancers [27], [32]. Various studies have documented that more than 90% of the p53 gene mutations in SCCHN in general are missense mutations, which are caused by a change in amino acid and a probable increase in the stability of the protein [28] which can be detected by immunohistochemical analysis due to stability of the protein [28], [29].

Studies have also strongly documented the correlation between the immunohistochemical overexpression of p53 protein and the presence of missense mutations within the p53 gene. Further, overexpression of p53 has been associated with poor survival in a number of studies [30], [31], [32].

In the present study, we have also found poor survival in patients where Cyclin-D1 and p53 were overexpressed as compared with moderate or negative expression, suggesting the possibility of occurrence of missense mutation in these patients and documenting the prognostic role of overexpressed p53 in OSCC. Thus, it is imperative to validate the IHC overexpressions of molecular markers with mutation analysis. This is also the drawback of the present study.

Therefore monitoring and manipulation of signal transduction pathway which forms the basis of treatment resistance to treatment may have important implications for the management of cancer. Direct targeting and inhibition of this pathway may increase radiosensitivity by antagonizing the radiation induced cellular defense mechanisms especially in tumors that have activated the PI3-K/AKT cascade. More importantly, specific targeting of this pathway in combination with radiotherapy or chemotherapy may enhance tumor control by antagonizing cellular defense in response to treatment.

Although many markers have been studied and have given new understanding of cancer pathogenesis and progression as a potential contributor to multistep process of oncogenesis they are not yet ready to be used as prognostic significance in routine clinical investigative and therapeutic procedures in patients with these tumors. Tumor stage, patient's age and performance status still remains the basis for therapeutic decisions. In view of this further studies are needed urgently to understand and delineate more specific and sensitive markers aiding in tumor diagnosis, prediction and prognostication of treatment modality to enhance the magnitude of treatment outcome in terms of response and survival of patients with these tumors.

## 5 Conclusion

The expressions of Cyclin-D1 and p53 and coexpressions of Cyclin-D1, EGFR and p53 may serve as prognostic markers in patients of locally advanced oral squamous cell carcinoma. The findings of molecular marker expressions may need further validations of mutation analysis.

## Conflict of interest

The authors declare that they have no conflict of interests.

## Figures and Tables

**Table 1:** Demographic and clinico-pathological characteristics of OSCC patients.

Characteristics	No. of OSCC patients (n = 97)	OSCC patients (%)
Age (years)		
≤ 40 years	28	28.9%
41–60 years	46	47.4%
> 60 years	23	23.7%
Sex		
Females	19	19.6%
Males	78	80.4%
Site of lesion		
Alveolus	15	15.5%
Buccal mucosa	24	24.7%
Cheek	7	7.2%
Hard palate	13	13.4%
Lip	9	9.3%
RMT	13	13.4%
Tongue	16	16.5%
Performance status		
Good	42	43.3%
Poor	55	56.7%
Histology		
Invasive squamous cell carcinoma	34	35.1%
Squamous cell carcinoma	63	64.9%
Grade		
Moderately differentiated (MD)	26	26.8%
Poorly differentiated (PD)	6	6.2%
Well differentiated (WD)	65	67.0%
Size		
T2	2	2.1%
T3	22	22.7%
T4	73	75.3%
Nodal status		
N0	32	33.0%
N1	32	33.0%
N2	33	34.0%
Stage		
III	28	28.9%
IV	69	71.1%

**Table 2:** Frequency distribution of molecular marker expressions of OSCC patients (n = 97).

Expression	Cyclin-D1 (n = 97) (%)	EGFR (n = 97) (%)	p53 (n = 97) (%)
Negative	13 (13.4)	7 (7.2)	14 (14.4)
Moderate positive	64 (66.0)	59 (60.8)	62 (63.9)
Strong positive	20 (20.6)	31 (32.0)	21 (21.6)
Total positive	84 (86.6)	90 (92.8)	83 (85.6)

**Table 3:** Correlation between Cyclin-D1, EGFR and p53 expressions in OSCC patients (n = 97).

Molecular marker	n	Cyclin-D1				EGFR			
Negative (n) (%)	Moderate positive (n) (%)	Strong positive (n) (%)	p value	Negative (n) (%)	Moderate positive (n) (%)	Strong positive (n) (%)	p value
EGFR									
Negative	7	1 (14.3)	6 (85.7)	0 (0.0)	0.634				
Moderate positive	59	9 (15.3)	37 (62.7)	13 (22.0)					
Strong positive	31	3 (9.7%)	21 (67.7)	7 (22.6)					
p53									
Negative	14	1 (7.1)	12 (85.7)	1 (7.1)	< 0.001	0 (0.0)	12 (85.7)	2 (14.3)	0.072
Moderate positive	62	11 (17.7)	45 (72.6)	6 (9.7)		7 (11.3)	32 (51.6)	23 (37.1)	
Strong positive	21	1 (4.8)	7 (33.3)	13 (61.9)		0 (0.0)	15 (71.4)	6 (28.6)	

**Table 4:** Association between molecular marker expressions and clinicopathological features in OSCC patients (n = 97).

Clinicopathological features	Cyclin-D1				EGFR				p53			
Negative (n = 13) (%)	Moderate positive (n = 64) (%)	Strong positive (n = 20) (%)	p value	Negative (n = 7) (%)	Moderate positive (n = 59) (%)	Strong positive (n = 31) (%)	p value	Negative (n = 14) (%)	Moderate positive (n = 62) (%)	Strong positive (n = 21) (%)	p value
Grade												
MD/PD	5 (38.5)	20 (31.3)	7 (35.0)	0.861	3 (42.9)	19 (32.2)	10 (32.3)	0.847	6 (42.9)	22 (35.5)	4 (19.0)	0.267
WD	8 (61.5)	44 (68.8)	13 (65.0)		4 (57.1)	40 (67.8)	21 (67.7)		8 (57.1)	40 (64.5)	17 (81.0)	
Tumor size												
T2/T3	4 (30.8)	16 (25.0)	4 (20.0)	0.780	1 (14.3)	18 (30.5)	5 (16.1)	0.259	6 (42.9)	12 (19.4)	6 (28.6)	0.165
T4	9 (69.2)	48 (75.0)	16 (80.0)		6 (85.7)	41 (69.5)	26 (83.9)		8 (57.1)	50 (80.6)	15 (71.4)	
Nodal status												
N0	6 (46.2)	24 (37.5)	2 (10.0)	0.041	2 (28.6)	23 (39.0)	7 (22.6)	0.281	9 (64.3)	19 (30.6)	4 (19.0)	0.017
N1/N2	7 (53.8)	40 (62.5)	18 (90.0)		5 (71.4)	36 (61.0)	24 (77.4)		5 (35.7)	43 (69.4)	17 (81.0)	
Stage												
III	6 (46.2)	19 (29.7)	3 (15.0)	0.151	2 (28.6)	20 (33.9)	6 (19.4)	0.351	6 (42.9)	16 (25.8)	6 (28.6)	0.445
IV	7 (53.8)	45 (70.3)	17 (85.0)		5 (71.4)	39 (66.1)	25 (80.6)		8 (57.1)	46 (74.2)	15 (71.4)	

**Table 5:** Association between molecular marker expressions and radiological response in OSCC patients (n = 97).

Molecular marker expressions	n	Radiological response			p value
CR (n) (%)	PR (n) (%)	NR (n) (%)
Cyclin-D1					
Negative	13	4 (30.8)	8 (61.5)	1 (7.7)	< 0.001
Moderate positive	64	24 (37.5)	36 (56.3)	4 (6.3)	
Strong positive	20	2 (10.0)	7 (35.0)	11 (55.0)	
EGFR					
Negative	7	3 (42.9)	3 (42.9)	1 (14.3)	0.014
Moderate positive	59	24 (40.7)	24 (40.7)	11 (18.6)	
Strong positive	31	3 (9.7)	24 (77.4)	4 (12.9)	
p53					
Negative	14	12 (85.7)	2 (14.3)	0 (0.0)	< 0.001
Moderate positive	62	16 (25.8)	44 (71.0)	2 (3.2)	
Strong positive	21	2 (9.5)	5 (23.8)	14 (66.7)	

CR = complete response, PR = partial response, NR = No response.

**Table 6:** Association between molecular marker expressions and coexpressions with overall survivals in OSCC patients (n = 97) using Cox regression analysis.

Molecular marker expressions and coexpressions	Univariate (unadjusted) Cox regression analysis		Multivariate (adjusted) Cox regression analysis	
OR (95% CI)	p value	OR (95% CI)	p value
Cyclin-D1				
Negative	Ref		Ref	
Moderate positive	0.56 (0.29–1.18)	0.136	0.80 (0.33–1.94)	0.624
Strong positive	0.59 (0.35–0.98)	0.041	0.98 (0.45–2.13)	0.954
EGFR				
Negative	Ref		Ref	
Moderate positive	0.58 (0.25–1.32)	0.191	0.55 (0.21–1.43)	0.217
Strong positive	1.14 (0.74–1.77)	0.548	1.45 (0.87–2.42)	0.156
p53				
Negative	Ref		Ref	
Moderate positive	0.39 (0.20–0.77)	0.007	0.86 (0.25–2.92)	0.802
Strong positive	0.61 (0.37–1.01)	0.053	1.12 (0.45–2.75)	0.809
Cyclin-D1 & EGFR				
Negative	Ref		Ref	
Moderate positive	0.67 (0.39–1.16)	0.154	0.63 (0.33–1.20)	0.160
Strong positive	0.81 (0.52–1.27)	0.359	1.19 (0.68–2.09)	0.540
Cyclin-D1 & p53				
Negative	Ref		Ref	
Moderate positive	0.43 (0.25–0.76)	0.028	0.69 (0.31–1.54)	0.360
Strong positive	1.58 (1.35–2.94)	0.003	1.90 (1.45–4.82)	0.046
EGFR & p53				
Negative	Ref		Ref	
Moderate positive	0.51 (0.30–0.86)	0.012	0.65 (0.30–1.41)	0.275
Strong positive	1.07 (0.68–1.69)	0.764	1.54 (0.87–2.72)	0.142
